# Interspecific Variation in the Inner Ear Maculae of Sharks

**DOI:** 10.1093/iob/obad031

**Published:** 2023-09-04

**Authors:** Derek J Sauer, Kara E Yopak, Craig A Radford

**Affiliations:** Leigh Marine Laboratory, Institute of Marine Science, University of Auckland, Leigh 0985, New Zealand; Department of Biology and Marine Biology and the Center for Marine Science, University of North Carolina Wilmington, Wilmington, NC 28409, USA; Leigh Marine Laboratory, Institute of Marine Science, University of Auckland, Leigh 0985, New Zealand

## Abstract

There is well-documented diversity in the organization of inner ear hair cells in fishes; this variation is thought to reflect the differing functional requirements of species across a range of ecological niches. However, relatively little is known about interspecific variation (and its potential ecological implications) in the number and density of inner ear hair cells in elasmobranchs (sharks, skates, and rays). In this study, we quantified inner ear hair cells in the saccule, lagena, utricle, and macula neglecta of 9 taxonomically and ecologically distinct shark species. Using phylogenetically informed comparative approaches, sharks that feed in the water column had significantly greater hair cell density and total number of hair cells in the lagena and macula neglecta (i.e., vertically oriented maculae) compared to species that feed primarily on the seafloor. In addition, sharks within Carcharhinidae seemingly possess a specialized macula neglecta compared to other shark species. Overall, findings suggest that, similar to bony fishes, there is considerable variation in hair cell organization of shark inner ears, which may be tied to variation in ecology and/or specialized behaviors between different species.

## Introduction

The attraction of free swimming sharks to certain underwater sounds was documented over 60 years ago (Nelson and Gruber 1963; Richard 1968; Myrberg et al. 1969) and, as a result, many subsequent studies endeavored to describe the auditory system of sharks and other elasmobranchs (reviewed by Chapuis and Collin 2022). Elasmobranchs (sharks, skates, and rays), unlike many bony fishes, do not possess a swim bladder or other gas-filled structure; the inner ears comprise the entirety of their auditory system. Each inner ear is composed of three semicircular canals (and their associated ampullae) and four end organs: three otoconial organs (the saccule, lagena, and utricle) and a non-otoconial end organ termed the macula neglecta. The macula neglecta, while diminutive in many fishes, is uniquely well developed in elasmobranchs and thought to serve an expanded role in their auditory processing (Tester et al. 1972; Fay et al. 1974; Corwin 1981a), specifically in the context of directional hearing (Corwin 1989; Myrberg 2001). However, its precise functional role in the auditory system of elasmobranchs remains unclear.

The semicircular canals are generally considered to be motion and body position sensors, while the end organs serve as auditory sensors, in addition to contributing to vestibular input (Platt and Popper 1981; Ladich and Popper 2004; Schulz-Mirbach et al. 2019). Each end organ within the inner ear houses a sensory epithelium (macula) of hair cells (HCs), which serve as mechano-electric transducers for vibrational stimuli. The end organs of all fishes function as particle motion detectors, converting differential movement between the denser otoconia (or a gelatinous cupula in the macula neglecta) and the sensory HCs into an electrical signal that is interpreted by the nervous system (De Vries 1950; Flock 1971; Hudspeth 1983). An individual HC within the inner ear is morphologically and physiologically polarized, with a graded decrease in response to stimuli that approach further from an axis of peak sensitivity (termed orientation) (Flock and Wersäll 1962; Hudspeth and Corey 1977). While each HC has its own orientation, the HCs in the inner ears of fish are divided into distinct groups that share the same orientation, an organizational layout thought to help enable directional hearing in fishes (Enger et al. 1973; Hawkins and Sand 1977; Popper et al. 1988).

**Fig. 1.**
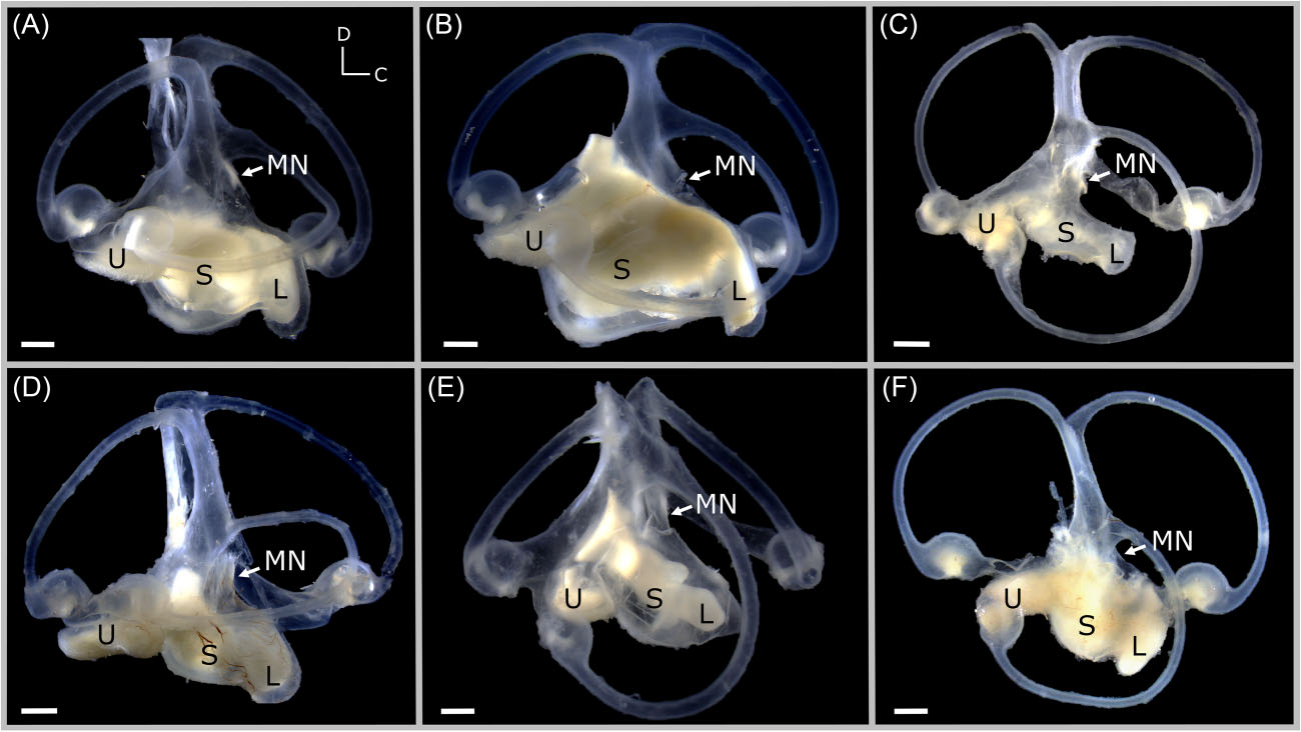
Photographs of the left inner ear of *G. galeus* (A), *M. lenticulatus* (B), *I. oxyrinchus* (C), *S. zygaena* (D), *C. isabellum* (E), and *A. vulpinus* (F), illustrating diversity in gross morphology of elasmobranch inner ears. S = saccule, L = lagena, U = utricle, MN = macula neglecta. Scale bars = 1 mm. All photographs have the same orientation. D = dorsal, C = caudal.

Although there is a large body of foundational research on inner ear morphology in elasmobranchs (see reviews by Chapuis and Collin 2022; Mickle and Higgs 2022), comparative anatomical studies between species are scarce. All elasmobranchs (and nearly all jawed fishes) are thought to share the same generic inner ear structures, but there is considerable variation between species in the shape and size of different components of the inner ear, including the semicircular canals and various end organs (e.g., Corwin 1978; Retzius 1881; Evangelista et al. 2010) (see Fig. 1). Previous work has proposed that this morphological diversity might reflect a combination of phylogenetic and ecological factors, such as feeding strategy and habitat (Corwin 1978; Evangelista et al. 2010), though this has yet to be empirically tested. In teleosts, variation in inner ear morphology has similarly been suggested to reflect different hearing and swimming requirements, primary habitat, and life history traits of different species (Platt and Popper 1981; Schellart and Popper 1992; Popper and Lu 2000). While differences in gross anatomy of the elasmobranch inner ear suggest that there are likely differences in HC organization (e.g., HC density, number, and orientation), as has been documented in teleost fishes (Popper 1977; Popper and Coombs 1982; Deng et al. 2013), these traits have only been examined in a few elasmobranchs to date. The total number of HCs was found to vary among two rays (Atlantic torpedo *Torpedo nobiliana* and bat ray *Myliobatis californica*) and four sharks (dusky smooth-hound *Mustelus canis*, tawny nurse shark *Nebrius ferrugineus*, broadnose sevengill *Notorynchus cepedianus*, and gray reef shark *Carcharhinus amblyrhynchos*) (Corwin 1978); however, these data are limited to assessment of HCs in the macula neglecta and do not account for differences in body size. Corwin (1981b) also qualitatively compared HC density in the macula neglecta from a few individuals of *C. falciformis* (silky shark) and *C. melanopterus* (blacktip reef shark), and suggested that HC density was the same in juveniles and adults of both species. In contrast, more recently, Sauer et al. (2022a,b) characterized variation in HC number, density, and orientation groups throughout ontogeny in *Galeorhinus galeus* (school shark) (Sauer et al. 2022a) and *Cephaloscyllium isabellum* (New Zealand carpet shark) (Sauer et al. 2022b), demonstrating considerable intraspecific variation across all four maculae throughout life in both species. Still, our understanding of interspecific variation in HC number and density, including potential drivers for this variation, is very limited.

While the functional significance of HC variation is unclear, the inner ear HCs of fishes are the structural basis for the auditory and vestibular systems, and thus may confer variation in auditory capabilities. Indeed, the number of HCs and/or HC density in the inner ear have been correlated to auditory sensitivity within several fishes (Corwin 1983; Coffin et al. 2012; Lu and Desmidt 2013; Wang et al. 2015), including elasmobranchs, where Corwin (1983) reported increased vibrational sensitivity in the macula neglecta of *Raja clavata* (thornback ray) with a greater absolute number of HCs. While more data are required to determine whether these findings are consistent throughout the clade, a comparative assessment of variation in HC organization across a range of species may provide a critical first step toward understanding differences in auditory and/or vestibular specialization in elasmobranchs. Therefore, this study characterized HC organization (area of the maculae, density of HCs, and total number of HCs) for each macula in nine different species of sharks. Across species, patterns of scaling were calculated and compared within and between each of the four inner ear end organs to assess whether variation in HC organization traits can be attributed to ecological differences between species.

## Materials and methods

### Fish collection

Individuals from nine species of sharks, *Alopias vulpinus* (common thresher), *Carcharhinus brachyurus* (bronze whaler), *Cephaloscyllium isabellum* (New Zealand carpet shark), *Galeorhinus galeus* (school shark), *Isurus oxyrinchus* (shortfin mako), *Mustelus antarcticus* (gummy shark), *Mustelus lenticulatus* (spotted estuary smooth hound), *Sphyrna zygaena* (smooth hammerhead), and *Squalus griffini* (northern spiny dogfish), were opportunistically obtained from local commercial and recreational fishers in Leigh, New Zealand. Total length (to the nearest 0.1 cm), body mass (to the nearest 0.1 kg), and sex were recorded from fresh, unfixed samples (Table 1). In instances where body mass could not be measured, it was estimated using published length–weight relationships (Froese and Pauly 2000). The otic capsules were removed from each individual and immersion-fixed in 4% paraformaldehyde in 0.1 M phosphate buffer. Samples were post-fixed for a minimum of 7 days (maximum of 16 weeks) before the inner ears were extracted.

**Table 1.** Total length (cm) and body mass (kg) range measurements from individuals of the nine shark species examined in this study, as well as the ecological categories that each species was assigned.

Species (abbr.)	Common name	n	Sex	Total length (cm)	Body mass (kg)	Feeding zone	Lifestyle
*Alopias vulpinus* (AV)	Common thresher	2	1M, 1U	148 – 152	5.3 – 5.7*	Water column	Pelagic
*Carcharhinus brachyurus* (CB)	Bronze whaler	2	2F	73 – 78	2.4 – 2.8	Water column	Benthopelagic
*Galeorhinus galeus* (GG)	School shark	3	1F, 2M	48–62	0.6 –0.7	Water column	Benthopelagic
*Isurus oxyrinchus* (IO)	Shortfin mako	2	1F, 1M	100–121	7.1 – 12.0*	Water column	Pelagic
*Sphyrna zygaena* (SZ)	Smooth hammerhead	3	2F, 1M	117– 144	6.7 – 11.8*	Water column	Pelagic
*Cephaloscyllium isabellum* (CI)	New Zealand carpet shark	3	1F, 2M	49–62	0.5 – 1.0	Benthos	Benthic
*Mustelus lenticulatus* (ML)	Spotted estuary smooth-hound	3	2M, 1U	54–73	0.5 – 1.4	Benthos	Benthopelagic
*Mustelus antarcticus* (MA)	Gummy shark	3	1F, 2M	78–95	1.5 – 3.9	Benthos	Benthopelagic
*Squalus griffini* (SG)	Northern spiny dogfish	3	1F, 2M	90–106	2.9 – 6.4	Benthos	Benthopelagic

The numbers of males and females are indicated with “M” and “F,” respectively, and individuals of unknown sex are represented with “U.” Species abbreviation code is listed in parentheses beside each species. Asterisks (*) indicate mass values that were based on published length/weight relationships (Froese and Pauly, 2000).

### Fluorescence microscopy and imaging

The four maculae (saccule, lagena, utricle, and macula neglecta) within the inner ear end organs were isolated and stained in 5 µL Oregon Green 488 Phalloidin (Invitrogen) mixed with 200 µL of phosphate buffer solution (1:40 dilution). Phalloidin selectively binds to the actin within HCs, enabling visualization of individual HCs (Lu and Popper 1998). Maculae were stained for 25 min, rinsed in phosphate buffer, and then mounted onto a glass microscope slide using Prolong Glass Antifade Mountant (Invitrogen), with the apical surface of the HCs facing upward.

Maculae were observed and photographed with a Nikon ECLIPSE Ni-U microscope equipped with an Intensilight C-HGFI epi-fluorescence illuminator, FITC filter set (excitation 465–495 nm), and Nikon DS-Qi2 camera. The NIS-BR Elements imaging software was used to image and digitally measure the maculae, as well as count HCs. Macular area measurements were acquired by stitching several 4×/0.13 N.A. objective images together to capture the entire macula, and HC counts were performed on 20×/0.50 N.A. objective images. HC orientations were determined throughout each macula from 40×/0.75 N.A. objective captures. Macular area measurements were not corrected for shrinkage due to fixation. HCs were defined as individual bundles of stereocilia and thus excluded any cells without stereocilia (e.g., early developing HCs).

### Morphological analysis

Hair cell density (HCD) in the otoconial organs was estimated using a stereological approach (following Sauer et al. 2022b), whereby HCs were imaged and counted in three non-overlapping quadrats along the length of each macula (one quadrat each in the rostral, medial, and caudal sections of the maculae). These quadrats were consistently placed at approximately 25, 50, and 75% the total length of the macula. Quadrats were scaled in size to allow for inclusion of the full width of the macula (both the central and marginal zones) and counting of approximately 10% of the total macular area. HCD was calculated in each quadrat by dividing the number of HCs (including those partially within the quadrat) by the area of the quadrat (HCs/µm^2^). HCD from the three quadrats was then averaged to estimate overall HCD for each macula, which was extrapolated to the total macular area to estimate the total number of hair cells (HCT) within each macula.

Since the macula neglecta is comprised of two separate patches of HCs in sharks (see Fig. 3) and is generally much smaller in absolute size than the other maculae, HCD in this macula was calculated using two quadrats (which were scaled in size as described above) placed along the length of each patch of the macula (four quadrats total). As preliminary *t*-tests indicated no significant differences in HCD between the two patches of the macula neglecta, the two separate epithelia were treated a single macula. HCT was estimated by extrapolating the average HCD from the four quadrats to the total area (both patches) of the macula.

**Fig. 2.**
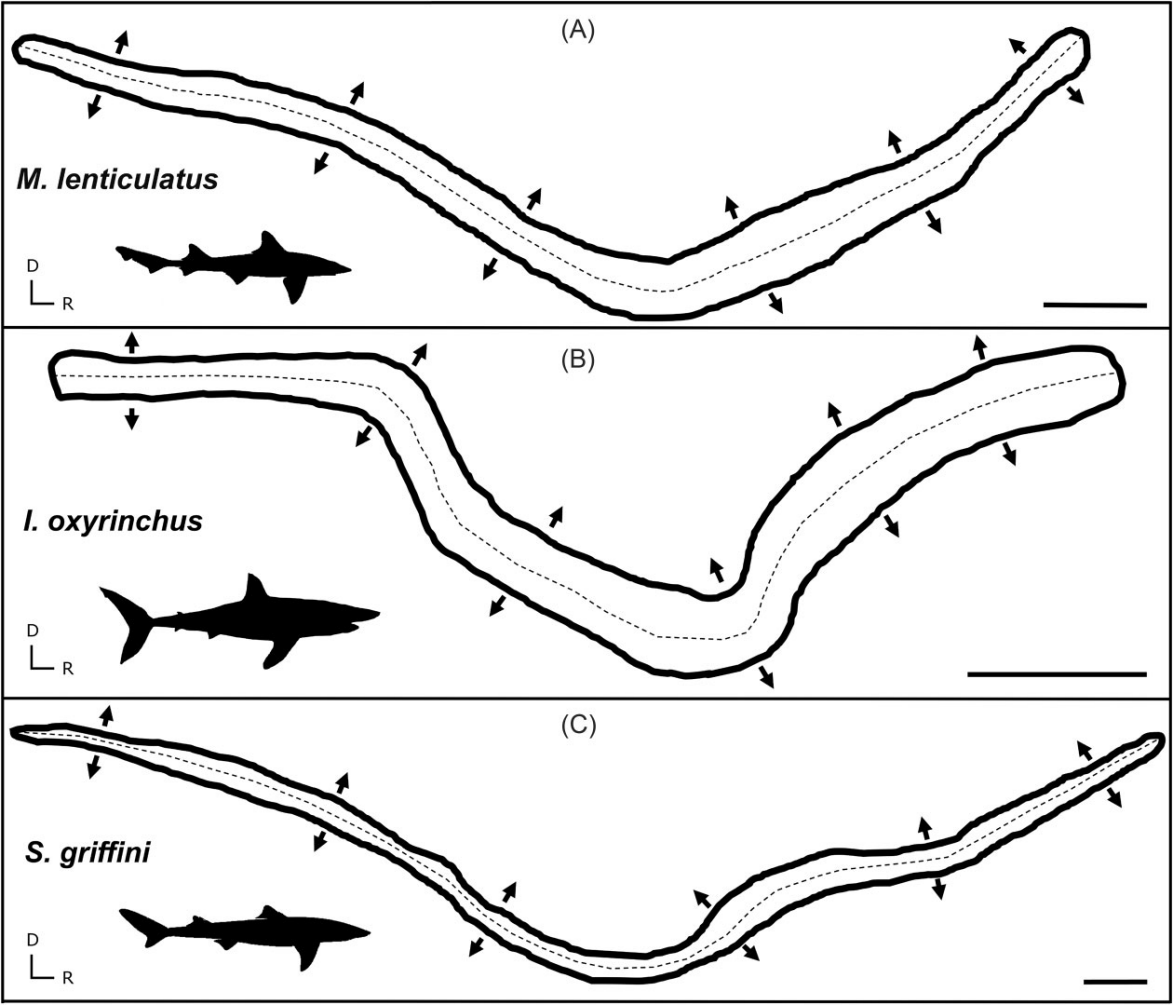
Tracings of the saccular maculae from three representative shark species, *M. lenticulatus* (A), *I. oxyrinchus* (B), and *S. griffini* (C), demonstrating the similar shape of the saccule and similar HC orientations (arrows indicate orientation) between species. Note that length and width should not be directly compared, as scales differ. Scale bars = 1000 µm. D = dorsal, R = rostral.

**Fig. 3.**
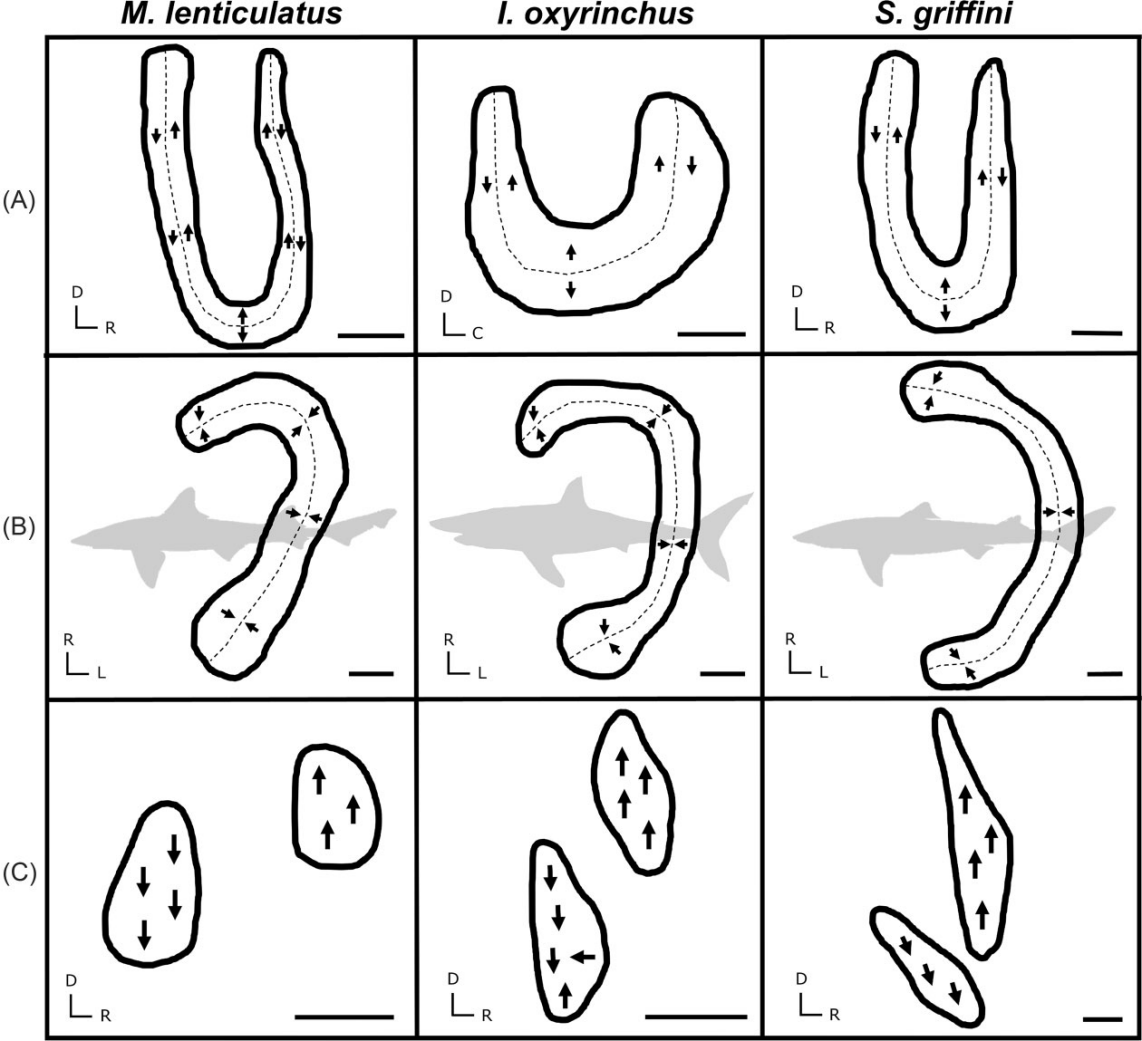
Tracings of the maculae from the lagena (A), utricle (B), and macula neglecta (C) in *M. lenticulatus, I. oxyrinchus*, and *S. griffini*, illustrating the general similarity of the three maculae shapes and HC orientations (arrows indicate orientation) between species. Note that length and width should not be directly compared, as macula shapes are not to scale with each other. Scale bars = 500 µm. D = dorsal, R = rostral, L = lateral.

Qualitative measurements of HC orientations were obtained from within each quadrat, as well as from additional areas in between quadrats and at the terminal ends of the maculae (to ensure HC orientation patterns were consistent throughout the entire macula). Each macula within the inner ear end organs was assigned to an “orientation” group based on how the orientations of its HCs lie in three-dimensional space. The lagena and macula neglecta were categorized as “vertical,” the utricle as “horizontal”, and the saccule as “mixed” (Platt and Popper 1981; Popper et al. 1988; Rogers et al. 1988).

### Statistical analyses

In order to assess interspecific scaling relationships between body size (total length, TL) and hair cell organization, data from individual sharks were averaged to obtain a mean value for each species (Table 1). In general, to limit allometric bias, all specimens were juveniles; individuals of the same species were of similar body sizes and included both males and females (if possible) to reduce the effect of intraspecific and sex-based variation on the species means used in comparative analyses. The means from each species were log_10_-transformed after corrected Akaike information criterion (AICc) scores across three candidate models (linear, log_10_-transformed, and square-root transformed data) indicated log_10_-transformed as the best-fitting model (Cavanaugh 1997; Burnham and Anderson 2002). (Note: raw data range values are located in Table S2.) Due to the statistical limitations of using only nine species means for phylogenetic analyses, a second dataset, which included measurements from two size-matched individuals of each species, was also utilized in subsequent analyses to increase statistical power.

To allow for results to be interpreted in relation to ecological factors, each species was assigned to a broad “feeding zone” category (Table 1). These zones were based on the feeding behaviors described in Corwin (1978), whereby species that generally prey upon invertebrates near the seafloor were categorized as “benthos,” and species that feed primarily by catching fish in open water were categorized as “water column.” For qualitative ecological comparisons, each species was also assigned to a primary lifestyle category: benthic (living on the bottom), benthopelagic (living near the bottom), and pelagic (living in the water column), based on published ecological literature (Musick et al. 2004; Yopak 2012).

Since generalized least squares (GLS) regression does not account for phylogenetic relationships, it will often overestimate the extent of a correlation and result in Type I errors (reviewed in Freckleton 2009). Therefore, interspecific scaling relationships between TL and HC organization traits were assessed using a phylogenetic GLS (pGLS) approach in the CAPER package in R (Orme et al. 2013), with total length as the independent variable (trait ∼ TL) (model 1). A phylogenetic tree was created by trimming a comprehensive molecular tree from Stein et al. (2018) down to the nine species examined in this study. As *C. isabellum* and *S. griffini* were not included in the larger tree, we assumed these species to be monophyletic with two closely related species (*Cephaloscyllium ventriosum* and *Squalus acanthias*, respectively).

The interspecific scaling relationships between TL and macular area, HCD, and HCT in each end organ were assessed using pGLS (model 1). We then tested an additional candidate pGLS model (model 2), which included feeding zone as an additional explanatory variable (trait ∼ TL + feeding zone). AICc values were used to identify the most parsimonious model, with the lowest AICc score indicating the best fit (Lavin et al. 2008) and differences in corrected Akaike values (ΔAICc) within 2 units also considered as having substantial support (Burnham and Anderson 2002).

For each HC organization trait, phylogenetically corrected residuals were obtained from model 1 and standardized to allow for relative (size-independent) comparisons between species and between different maculae. Residuals were analyzed with phylogenetic analysis of variance (ANOVA), using “phylANOVA” in the GEIGER package in R (Harmon et al. 2008), to compare macular area, HCD, and HCT between different feeding zones. However, as feeding zones failed to explain a significant amount of variation in phylogenetic residuals in any of the end organs (Table S1), to increase statistical power, we ran a GLS regression using two individuals from each species (model 3). We then obtained standardized residuals from each individual of the GLS regression, which were then used to analyze differences in HC organization across species. These residuals were compared between feeding zones and maculae orientation groups, using one-way ANOVAs (or Welch's ANOVA in instances where residuals had unequal variances).

## Results

### Gross observations

**Fig. 4.**
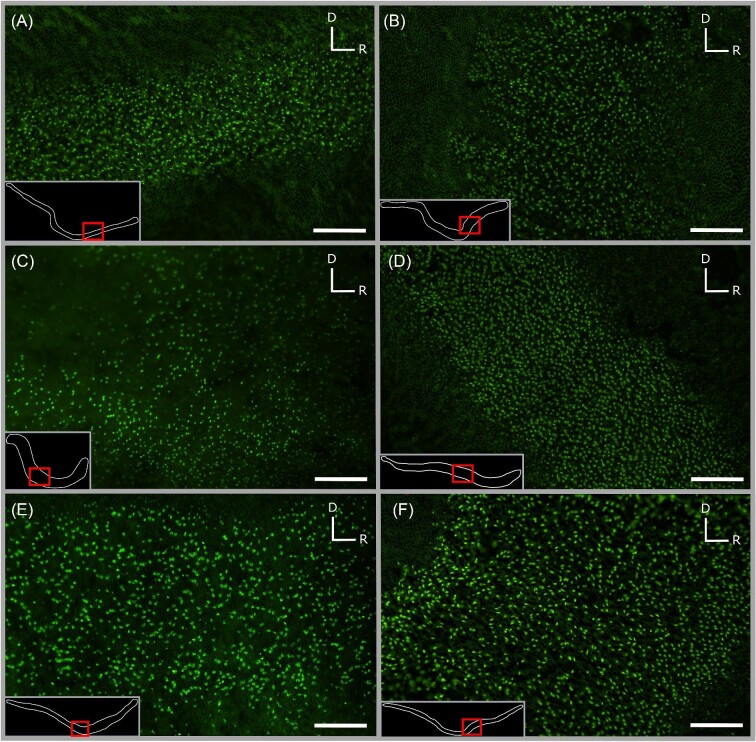
Fluorescent micrographs (20× objective) of saccular hair cells from six shark species examined in this study, *C. isabellum* (A), *I. oxyrinchus* (B), *S. zygaena* (C), *G. galeus* (D), *M. lenticulatus* (E), and *S. griffini* (F), illustrating visible variation in hair cell density and saccular width. Note that insets are not to scale and serve only to contextualize micrographs. Scale bars (100 µm) in micrographs are equivalent to allow for direct comparison. D = dorsal, R = rostral.

**Fig. 5.**
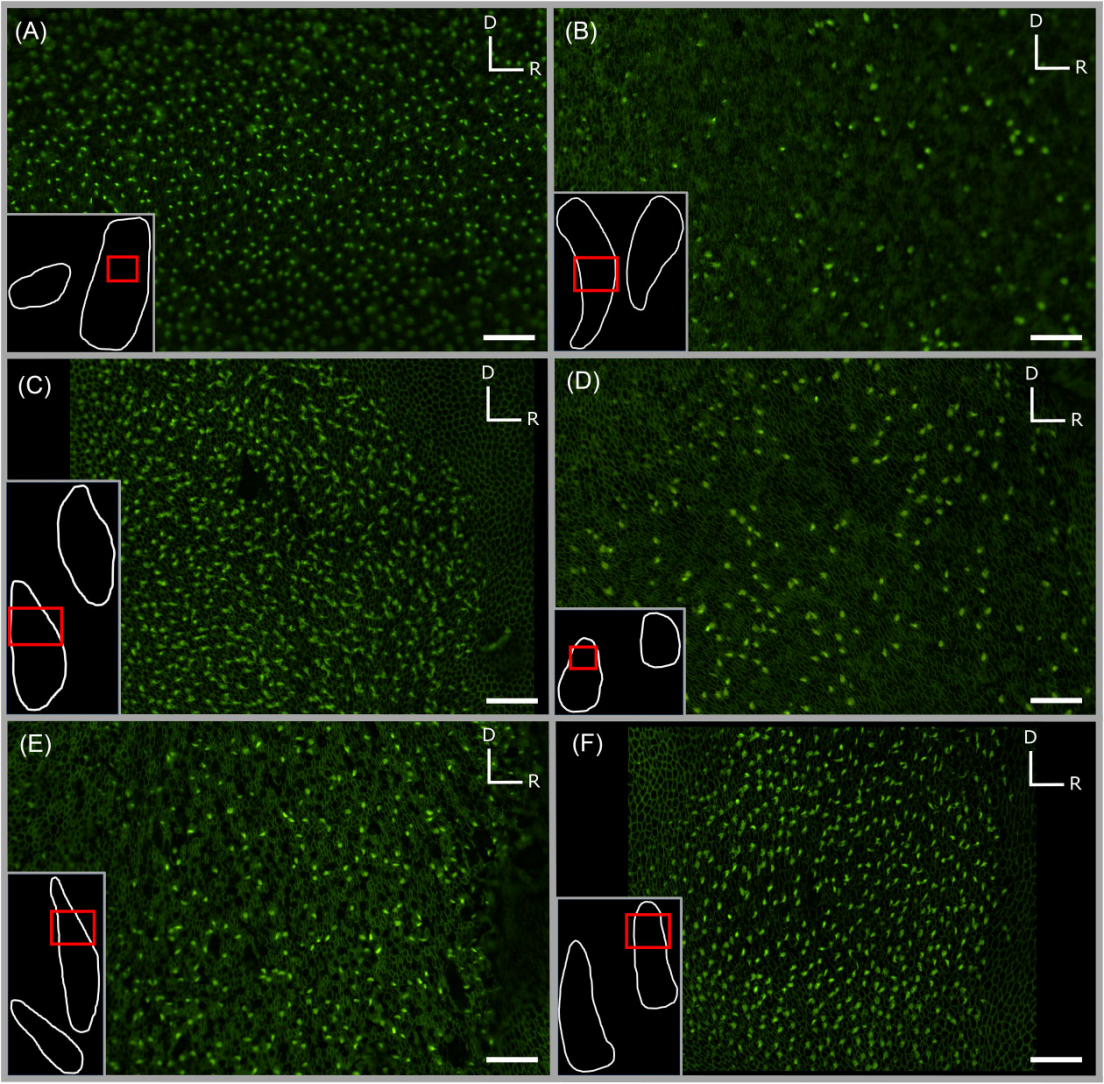
Fluorescent micrographs (20× objective) of hair cells in the macula neglecta of six shark species examined in this study, *Ca. brachyurus* (A), *S. zygaena* (B), *I. oxyrinchus* (C), *M. lenticulatus* (D), *S. griffini* (E), and *G. galeus* (F), illustrating visible variation in hair cell density. Note that insets are not to scale and serve only to contextualize micrographs. Scale bars (50 µm) in micrographs are equivalent to allow for direct comparison. D = dorsal, R = rostral.

The general shape of each of the four maculae was similar in all shark species examined. Generally, the saccule exhibited an elongated “v” shape, the lagena a “u” shape, and the utricle a “c” shape. The macula neglecta had two separate, ovular epithelia in close proximity to each other (Figs. 2 and 3). These generic maculae shapes were observed in all species. Similar HC orientation groups were also observed within each macula across species. The saccule and lagena contain two groups of HCs with opposing (180° difference) orientations that face away from a midline that bisects the maculae, while the utricle contains two groups of HCs with opposing orientations that face *toward* a midline (Figs. 2 and 3). The macula neglecta is composed of two separate patches of HCs with opposing orientations (Fig. 3). Most notably, we observed deviation from these generic patterns in the macula neglecta of *I. oxyrinchus*, whereby the posterior patch contained HCs with a variety of orientations instead of all HCs oriented in the ventral direction (Fig. 3). There were visible differences between species in the absolute size (area) and apparent HCD of the maculae in each of the end organs (Figs. 4 and 5).

### Allometric scaling

**Fig. 6.**
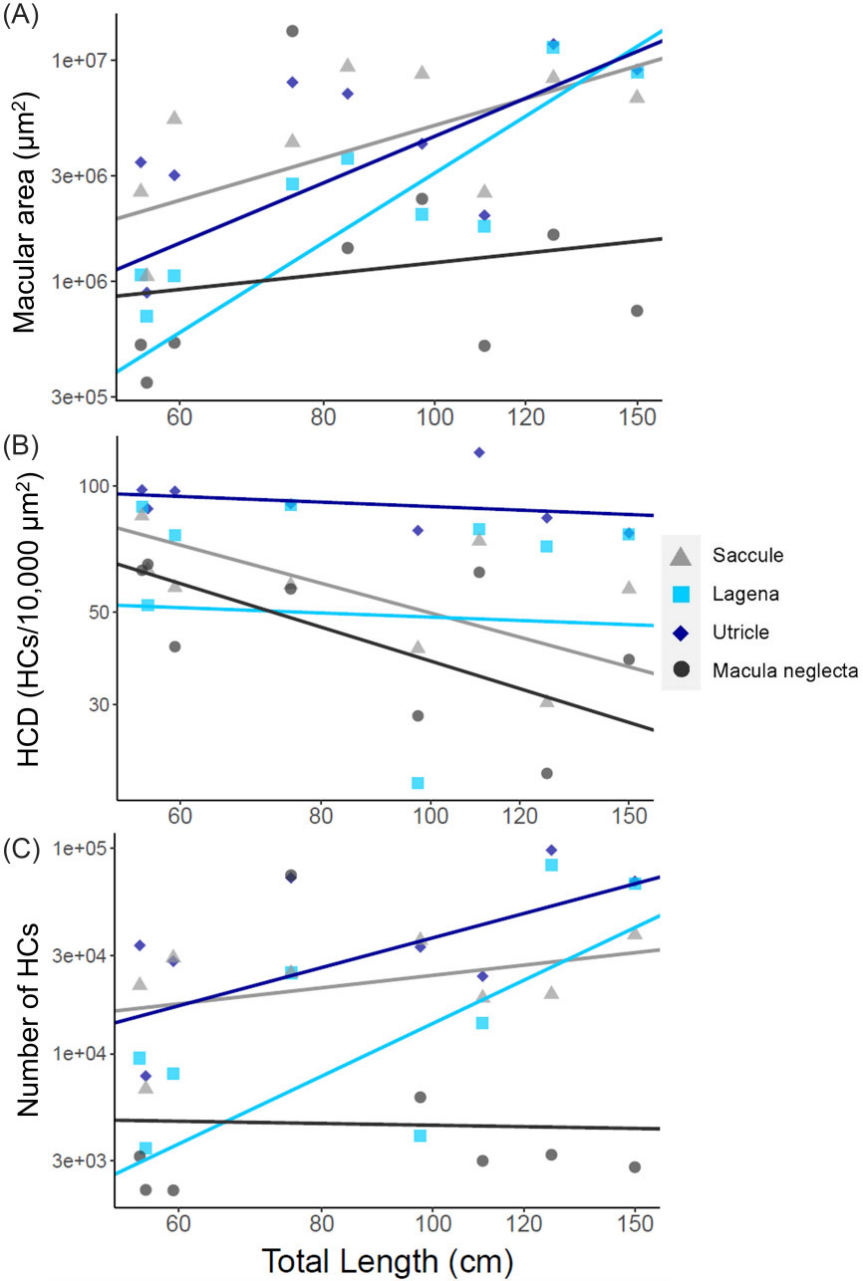
(A) Interspecific scaling of macular area and total length (logarithmic axes) in each of the four inner ear maculae of nine species of sharks from pGLS models. Saccule = 1.53× + 6.35 (*P* = 0.001, *r*^2^ = 0.78); Lagena = 3.25× −0.01 (*P* < 0.001, *r*^2^ = 0.95); Utricle = 2.18× + 2.30 (*P* = 0.001, *r*^2^ = 0.83); Macula neglecta = 0.55× + 4.99 (*P* = 0.651, *r*^2^ = 0.03). (B) Interspecific scaling of hair cell density (HCD) and total length (logarithmic axes) in the four inner ear maculae of nine species of sharks from pGLS models. Saccule = −0.73× + 3.16 (*P =* 0.033, *r*^2^ = 0.56); lagena = –0.10× + 1.89 (*P* = 0.800, *r*^2^ = 0.01); utricle = –0.11× + 2.17 ( = 0.480, *r*^2^ = 0.09); macula neglecta = –0.83× + 3.25 (*P* = 0.051, *r*^2^ = 0.49). (C) Interspecific scaling of the total number of HCs with total length (logarithmic axes) in each of the four inner ear maculae of nine species of sharks, from pGLS models. Saccule = 0.63× + 3.13 (*P* = 0.264, *r*^2^ = 0.20); lagena = 2.65× –1.15 (*P* = 0.006, *r*^2^ = 0.74); utricle = 1.50× + 1.58 (*P* = 0.035, *r*^2^ = 0.55); macula neglecta = −0.09× + 3.83 (*P* = 0.945, *r*^2^ = 0.00).

Using species’ means as data points (model 1), macular area scaled positively with body size (TL) in the saccule (*F*_1,7_ = 25.44, *P* = 0.001, *r*^2^ = 0.78), lagena (*F*_1,7_ = 137.20, *P* < 0.001, *r*^2^ = 0.95), and utricle (*F*_1,7_ = 34.96, *P* = 0.001, *r*^2^ = 0.83). There was, however, no significant relationship between macular area and body size in the macula neglecta (*F*_1,7_ = 0.22, *P* = 0.651, *r*^2^ = 0.03) (Fig. 6A).

There was a significant negative relationship between TL and HCD in the saccule (*F*_1,6_ = 7.66, *P* = 0.033, *r*^2^ = 0.56), and a trend toward a negative relationship between TL and HCD in the macula neglecta (*F*_1,6_ = 5.87, *P* = 0.051, *r*^2^ = 0.49), but this was not significant. In contrast, HCD in the utricle (*F*_1,6_ = 0.57, *P* = 0.480, *r*^2^ = 0.09) and lagena (*F*_1,6_ = 0.07, *P* = 0.800, *r*^2^ = 0.01) did not scale significantly with TL (Fig. 6B).

HCT scaled positively with TL in the lagena (*F*_1,6_ = 17.02, *P* = 0.006, *r*^2^ = 0.74) and utricle (*F*_1,6_ = 7.41, *P* = 0.035, *r*^2^ = 0.55), but there was no significant relationship between TL and HCT in the saccule (*F*_1,6_ = 1.52, *P* = 0.264, *r*^2^ = 0.20) or macula neglecta (*F*_1,6_ = 0.01, *P* = 0.945, *r*^2^ = 0.00) (Fig. 6C).

### Phylogenetically corrected residuals

**Fig. 7.**
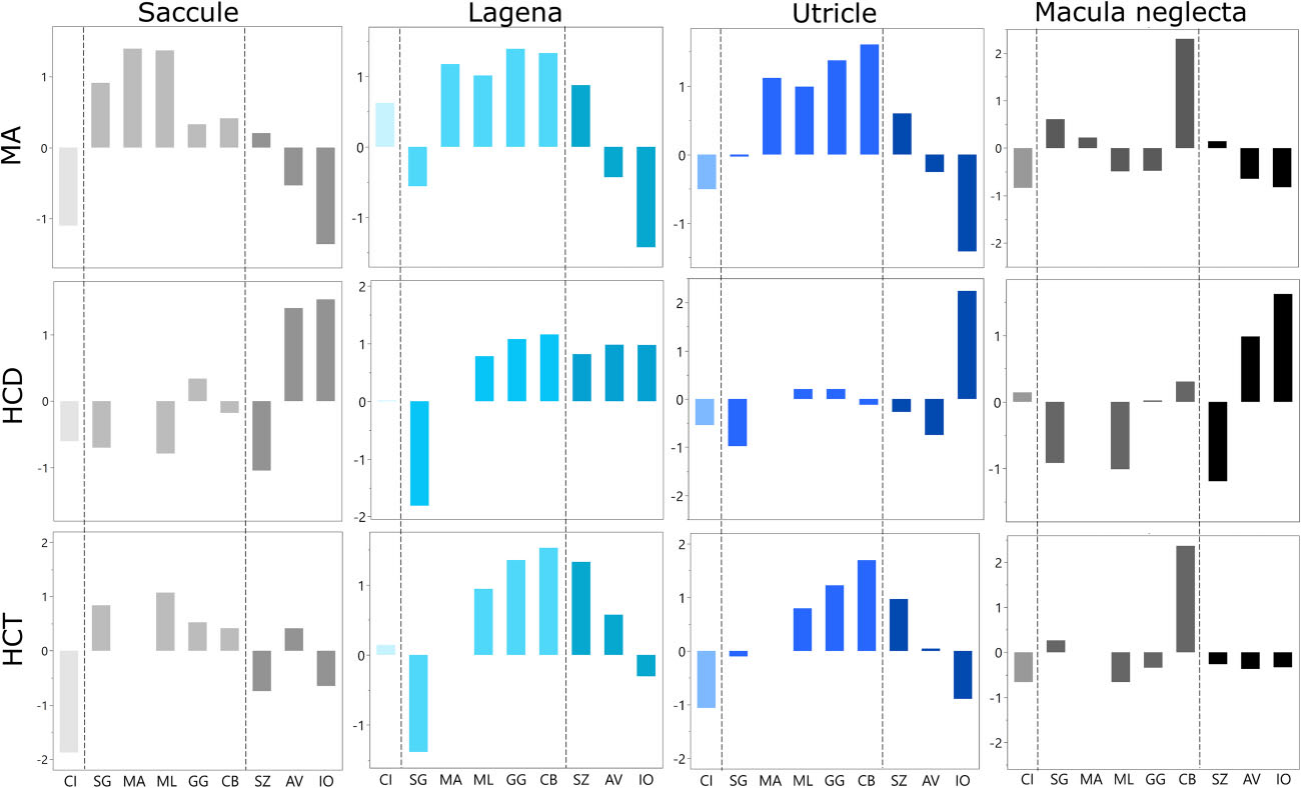
Phylogenetically corrected, standardized residuals predicted from body size (total length) for MA, HCD, and HCT in the saccule, lagena, utricle, and macula neglecta of nine species of shark, from pGLS models. In each panel, species are shaded according to lifestyle (lightest shade: benthic; mid-shade: benthopelagic; darkest shade: pelagic). For species abbreviations, see Table 1.

The largest saccular maculae were in *M. lenticulatus* (residual (*R*_sac_) = 1.4) and *M. antarcticus* (*R*_sac_ = 1.4), while *I. oxyrinchus* (*R*_sac_ = −1.4) and *C. isabellum* (*R*_sac_ = −1.1) had the smallest saccular maculae (Fig. 7). HCD residuals (*R*_HCD_) in the saccule was greatest in *A. vulpinus* (*R*_HCD_ = 1.4) and *I. oxyrinchus* (*R*_HCD_ = 1.4) and lowest in *S. zygaena* (*R*_HCD_ = −1.0) and *S. griffini* (*R*_HCD =_ −0.7). Although we were unable to obtain HCT measurements in *M. antarcticus, M. lenticulatus* had the highest HCT residuals (*R*_HCT_ = 1.1) in the saccule, with *C. isabellum* having the lowest saccular HCT (*R*_HCT_ = −1.9).

*Carcharhinus brachyurus* (*R*_lag_ = 1.3) and *G. galeus* (*R*_lag_ = 1.4) had relatively large lagenar maculae, while *I. oxyrinchus* (*R*_lag_ = −1.4) had a notably small lagenar macula. HCD in the lagena was greatest in *G. galeus* (*R*_HCD_ = 1.1) and *C. brachyurus* (*R*_HCD_ = 1.2) and lowest in *S. griffini* (R_HCD_ = −1.8), while lagenar HCT was also greatest in *C. brachyurus* (*R*_HCT_ = 1.5) and *G. galeus* (*R*_HCT_ = 1.4) and lowest in *S. griffini* (*R*_HCT_ = −1.4) (Fig. 7).

The utricular maculae in *C. brachyurus* (*R*_utr_ = 1.6) and *G. galeus* (*R*_utr_ = 1.4) were relatively large, while *I. oxyrinchus* had the smallest utricular macula (*R*_utr_ −1.4). *Isurus oxyrinchus* had considerably high HCD in the utricle (*R*_HCD_ = 2.2), while *S. griffini* (*R*_HCD_ = −1.0) and *A. vulpinus* (*R*_HCD_ = −0.7) had relatively low HCD in the utricle (Fig. 7). HCT in the utricle was greatest in *C. brachyurus* (*R*_HCT_ = 1.7) and *G. galeus* (*R*_HCT_ = 1.2), and lowest in *C. isabellum* (*R*_HCT_ = −1.0) and *I. oxyrinchus* (*R*_HCT_ = −0.9).

The macula neglecta of *C. brachyurus* was extremely large (residual (*R*_MN_) = 2.3) compared with all other species (Fig. 7). In fact, the mean absolute size of the macula neglecta in *C. brachyurus* (13.54 mm^2^) was more than five-fold greater than the next closest species, *S. griffini* (2.36 mm^2^), despite *C. brachyurus* having the fourth smallest mean body size. In contrast, *C. isabellum* (*R*_MN_ = −0.8) and *I. oxyrinchus* (*R*_MN_ = −0.8) had the smallest macula neglectas. HCD in the macula neglecta was greatest in *I. oxyrinchus* (*R*_HCD_ = 1.6) and *A. vulpinus* (*R*_HCD_ = 1.0), and lowest in *S. zygaena* (*R*_HCD_ = −1.2) and *M. lenticulatus* (*R*_HCD_ = −1.0). Lastly, HCT in the macula neglecta was greatest in *C. brachyurus* (*R*_HCT_ = 2.4), and lowest in *C. isabellum* (*R*_HCT_ = −0.7) and *M. lenticulatus* (*R*_HCT_ = −0.7).

### Interspecific variation in macular area, HCD, and HCT

**Fig. 8.**
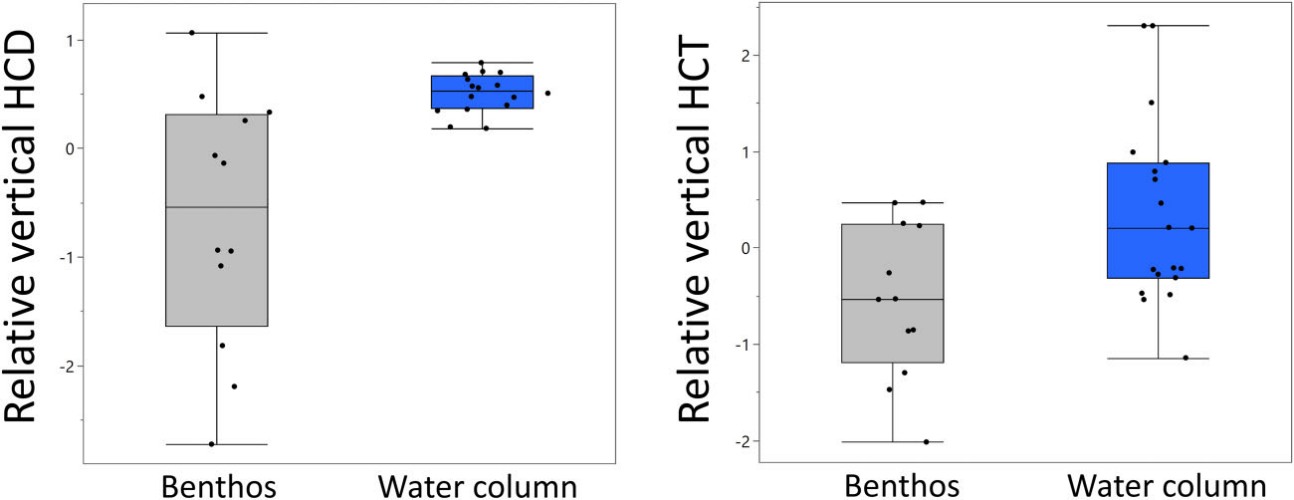
Comparison of standardized residuals, calculated from GLS models (model 3), of HCD and HCT from vertically oriented HCs (lagena and macula neglecta) between benthos and water column feeding shark species.

Phylogenetic ANOVA of standardized residuals from pGLS models (model 1) indicated no significant differences in macular area, HCD, or HCT between feeding zones in any of the maculae (Table S1). However, GLS standardized residuals, from a subset of data containing two size-matched individuals from each species (model 3), showed significant differences between feeding zones in lagenar HCD (*F*_1,14_ = 10.94, *P* = 0.005) and lagenar HCT (*F*_1,14_ = 6.76, *P* = 0.021). In addition, after grouping model 3 residuals based upon the orientation of the maculae (e.g., vertical vs. horizontal), HCD (*F*_1,29_ = 11.60, *P* = 0.002) and HCT residuals (*F*_1,29_ = 7.00, *P* = 0.013) from the vertically oriented maculae (lagena and macula neglecta) were significantly greater in water column feeding species compared to benthos feeding species (Fig. 8). However, there was no significant difference in macular area residuals in the vertically oriented maculae (*F*_1,33_ = 0.58, *P* = 0.451); there were also no significant differences in macular area, HCD, or HCT residuals between benthos and water column feeding species in the horizontally and mixed oriented macula (see Supplementary Material).

### Phylogenetic modeling

The scaling of macular area was best explained by model 1 (macular area ∼ TL) in all four maculae, although there was substantial support (ΔAICc <2) for model 2 (macular area ∼ TL + feeding zone) in the macula neglecta. The scaling of HCD was also best explained by model 1 (HCD ∼ TL), but there was substantial support for model 2 in the saccule (HCD ∼ TL + feeding zone) and lagena (HCD ∼ TL + feeding zone). Lastly, the scaling of HCT was best explained by model 1 (HCT ∼ TL) in all four inner ear maculae (Table 2).

**Table 2.** AICc scores and ΔAICc values (in parentheses) from pGLS model comparisons of macular area, HCD, and HCT in the saccule, lagena, utricle, and macula neglecta.

	**Saccule**		
	Macular area	HCD	HCT
Model 1 (trait ∼ TL)	1.1 (0.00)	−5.9 (0.00)	3.1 (0.00)
Model 2 (trait ∼ TL + feeding zone)	4.1 (3.02)	−4.2 (1.64)*	8.7 (5.6)

	**Lagena**		
Model 1 (trait ∼ TL)	−0.5 (0.00)	0.0 (0.00)	8.3 (0.00)
Model 2 (trait ∼ TL + feeding zone)	4.3 (4.79)	0.8 (0.80)*	10.7 (2.34)
	**Utricle**		
Model 1 (trait ∼ TL)	**4.6 (0.00)**	**−17.1 (0.00)**	**5.8 (0.00)**
Model 2 (trait ∼ TL + feeding zone)	9.3 (4.66)	−12.9 (4.14)	9.3 (3.43)
	**Macula Neglecta**		
Model 1 (trait ∼ TL)	**−5.9 (0.00)**	**−2.4 (0.00)**	**17.2 (0.00)**
Model 2 (trait ∼ TL + feeding zone)	−4.2 (1.64)*	−0.4 (2.05)	21.9 (4.68)

Model 1 was consistently the best-fitting model (as indicated by the lowest AICc score, emboldened); however, model 2 was also substantially supported (ΔAICc < 2) in some instances, which are indicated with an asterisk (*).

## Discussion

Differences in the organization of inner ear HCs in fishes may reflect variation in the relative hearing capabilities of different species (Corwin 1983; Coffin et al. 2012; Lu and Desmidt 2013; Wang et al. 2015; Lara et al. 2022). While there is known interspecific variation in the gross inner ear morphology of elasmobranchs (Corwin 1978; Retzius 1881; Evangelista et al. 2010), comparative data on HC organization within this group are lacking, and the extent of interspecific variation in HC number and density is largely unknown. In this study, we quantified macular area, HCD, and HCT in the inner ear maculae of nine different shark species. We aimed to determine whether variation in HC organization could be partially explained by differences in primary feeding zones of these species (on the benthos or in the water column), as had been previously proposed (Corwin 1978; Evangelista et al. 2010). While the general shapes and orientations of inner ear maculae appear to be primarily conserved across shark species, there were interspecific differences between species in relative macular area, HCD, and HCT in each of the four inner ear end organs. Overall, this study shows that, similar to gross inner ear morphology, there is considerable anatomical diversity in the sensory maculae of sharks, which may be associated with variation in ecology.

Intraspecifically, positive correlations between the size of inner ear maculae (and number of HCs) and body growth have been established within numerous fish species (Lombarte and Popper 1994; Higgs et al. 2003; Wang et al. 2015; Chaves et al. 2017; Lozier and Sisneros 2020), including several elasmobranchs (Corwin 1981b, 1983; Barber et al. 1985; Sauer et al. 2022a,b), owing to a continued growth of the inner ear throughout ontogeny. However, comparative datasets spanning a wide range of ecologically and taxonomically diverse species are lacking, and relatively little is known about interspecific variability between maculae in sharks. In the current study, macular area and HCT scaled positively with body size across nine shark species in the saccule, lagena, and utricle (though marginally not significant in the saccule). The lagena exhibited the steepest rate of allometric growth across species, with macular area and HCT scaling with body size at a rate approximately 1.5–2 times greater than all other end organs. In fact, lagenar area scales with hyperallometry (slope = 3.25), growing over 60% faster than body length. In other words, large-bodied sharks tend to possess relatively larger maculae (with a particularly large lagena), with a higher number of HCs. In contrast, macular area and HCT did not scale positively with body size in the macula neglecta. Although previous studies have documented positive scaling between body size and macula neglecta size throughout life in individual species (Barber *et al.* 1985; Corwin 1981b, 1983; Sauer et al. 2022a), the macula neglecta was not influenced by body size interspecifically, at least across the nine species examined here. In addition, HCD scaled negatively with body size across shark species in the saccule and macula neglecta. Intraspecifically, HCD often decreases throughout ontogeny in bony fishes (Popper and Hoxter 1984; Lombarte and Popper 1994; Chaves et al. 2017; Lozier and Sisneros 2020), although whether this relationship exists across a wide range of species is unknown. Future work should aim to determine if larger bodied fish species exhibit relatively low HCD, as variation in HC organization likely has functional implications.

Previous comparative work in elasmobranchs has shown considerable variation in morphological characteristics of other sensory systems (e.g., visual, olfactory, and electrosensory systems) (Kajiura 2001; Lisney and Collin 2007; Schluessel et al. 2008; Kempster et al. 2012; Ferrando et al. 2019), and similar variability in the size of brain regions that receive primary afferents from these systems (Yopak and Montgomery 2008; Kajiura et al. 2010; Yopak and Lisney 2012; Yopak et al. 2015). This variation is correlated with a range of ecological factors, including primary habitat, lifestyle characteristics, and diet (see Collin 2012; Collin et al. 2015; Meredith et al. 2022; Yopak 2022 for review). Despite the potential relationship between HCT and/or HCD and inner ear sensitivity in fishes (Corwin 1983; Coffin et al. 2012; Lu and Desmidt 2013; Wang et al. 2015; Lara et al. 2022), interspecific variation in these traits has rarely been considered in elasmobranchs. Corwin (1981b) reported anecdotal evidence that HCD in the macula neglecta of two reef shark species (*Ca. falciformis* and *Ca. melanopterus*) was similar, but this comparison was carried out in a small number of individuals that were very similar taxonomically and ecologically.

Across the nine species investigated here, there are notable differences in relative macular area, HCD, and HCT in each of the four inner ear end organs, which may be associated with functional differences. Corwin (1978) proposed that variation in the inner ear may reflect differences in feeding mode, with distinctions between species that feed on the benthos or are generally ambush predators (“non-raptorial”) and those in the water column that feed on active, mobile prey (“raptorial”). This was later elaborated by Evangelista et al. (2010), whereby four distinct groups were proposed and discussed in relation to feeding strategies. While differences in HC organization in this study did not fully conform to Corwin’s (1978) feeding mode hypothesis, there was significant interspecific variation in vertically oriented HCs that correlated with feeding zone. In particular, the lagena may represent a point of differentiation between sharks that feed in the benthos and those that feed in the water column. In elasmobranchs, the HCs within the lagena (and macula neglecta) are oriented in the vertical plane, where nearly all HCs are dorsally or ventrally oriented (Lowenstein et al. 1964; Platt and Popper 1981; Lovell et al. 2007; Sauer et al. 2022a,b). Since the orientation of each HC determines its physiological response to directional stimuli (Flock and Wersäll 1962; Hudspeth and Corey 1977), HCs oriented in the dorsal and ventral directions are most sensitive to vertically propagating stimuli. Species feeding in pelagic environments, with sources of acoustical disturbances occurring both above and below, could potentially benefit from increased sensitivity to vertical stimuli. Accordingly, water column-feeding species in this study (see Table 1) had greater relative HCD and HCT in the lagena and macula neglecta, compared to benthic feeding species. This difference appears to be largely driven by the lagena, as the macula neglecta alone did not exhibit significant differences in HCD and HCT between feeding zones. Interestingly, the macula neglecta of *I. oxyrinchus* exhibited some of the minimal variation detected in HC orientations. These findings merit further investigation given the small number of *I. oxyrinchus* individuals examined in this study and the suspected importance of the vertically oriented macula neglecta in elasmobranchs.

Species that actively find and catch prey in open water may gain a predatory advantage from increased sensitivity to vertically propagating stimuli, whereby selection for greater HCD and HCT in the lagena of these species is plausible. Although this study could not test for this due to small sample sizes, this hypothesis is anecdotally supported by the exceptionally high HCD in the lagena and macula neglecta of *I. oxyrinchus* and *A. vulpinus*, two pelagic, piscivorous species that feed primarily in the water column (Smith et al. 2008; Stevens 2008). However, further work is required to determine whether this pattern is consistent with other species, especially since *S. zygaena*, another pelagic species, showed the opposite trend. Importantly, juvenile *Sphyrna* (i.e., those in this study) primarily feed on benthic squids in coastal habitats, while adults typically prey on pelagic squids offshore (Smale 1991; Rojas et al. 2014; Estupiñán-Montaño et al. 2019). As macular area and HCT continue to increase throughout ontogeny (Sauer et al. 2022a,b), the trends seen here may not represent the adult condition in this species.

While the small sample sizes in the current study limited its statistical power to examine variation in the inner ear in relation to lifestyle characteristics (e.g., benthic, benthopelagic, and pelagic species), there were broad similarities in HC organization between sharks that share similar lifestyles (Fig. 7). For example, *I. oxyrinchus* and *A. vulpinus*, two pelagic species in this study, both possess relatively small maculae with relatively high HCD. Given sound propagates at high speed and extensive distances underwater (given variation in temperature, pressure, and salinity) (Urick 1979; Rogers and Cox 1988), particularly in unobstructed open ocean, it is possible that unique specializations in the inner ear are found in oceanic species to capitalize on these signals. In contrast, benthopelagic species consistently shared relatively large maculae with relatively high HCT, which could suggest the importance of acoustic signals in key demersal coastal habitats, such as coral and temperate reefs (Radford et al. 2008, 2011, 2014). Interestingly, *S. griffini* is both a notable outlier in its HC organization and the only deep sea species examined in this study; the extreme environment of the deep sea may lend unique adaptations of the auditory system, as in other aspects of the nervous system (e.g., Yopak and Montgomery 2008; Kempster et al. 2012; Yopak and Lisney 2012; Yopak et al. 2015). Lastly, *Ce. isabellum*, the only strictly benthic species in this dataset (i.e., spends most of its time resting on the seafloor), had relatively small maculae, low HCD, and low HCT, which may be indicative of reduced auditory capacity. Alternatively, it is possible that reduced sensory maculae in *C. isabellum* are related to the vestibular rather than the auditory system. Although highly speculative, given a close association with the substrate, and thus perhaps fewer proprioceptive demands than open water dwelling species, coupled with a sit and wait predation strategy (Horn 2016), vestibular requirements may be reduced in benthic species compared to more benthopelagic and pelagic sharks. The above predictions are based on very few species, and undoubtedly require much more examination before any trends can be established. At present, structure–function relationships and the relative importance of different sensory systems across elasmobranch fishes are unclear and additional data on macula size, HCD, and HCT across a wider range of species and in more individuals are required to understand the ecological drivers (and potential functional significance) of variation in the inner ear.

The macula neglecta has long been considered a vital component of auditory processing in sharks. The majority of research on shark audition, especially in the context of directional hearing (Popper and Fay 1977; Corwin 1989; Myrberg 2001), has focused on this structure, likely given its relatively large size, anatomical position (Tester et al. 1972; Corwin 1977), and remarkable sensitivity to vibrations (Lowenstein and Roberts 1951; Fay et al. 1974; Corwin 1981a). Previous studies on elasmobranchs have reported that this macula contains some of the largest populations of HCs observed in vertebrates (Corwin 1977), and increases substantially in size and HC number throughout ontogeny (Corwin 1981b, 1983; Barber et al. 1985; Sauer et al. 2022a). However, much of the research on the macula neglecta in sharks has been conducted in members of the family Carcharhinidae (Tester et al. 1972; Fay et al. 1974; Corwin 1977, 1981a,b), with very little data available on the macula neglecta in sharks outside this family. In one non-carcharhinid species, *Scyliorhinus canicula* (small-spotted catshark), the macula neglecta contained 500,000 “elongate structures” that are different from typical sensory HCs (Lovell et al. 2007). Thus, it is difficult to say whether reported features of the macula neglecta in carcharhinids are representative of all species within the clade or are unique to this group. Across representatives from seven families in this study, the macula neglecta was consistently the smallest macula in absolute area, and had relatively low HCD and HCT (compared to the other three maculae) in all non-carcharhinid species. Interestingly, the macula neglecta was the largest macula and contained the greatest number of HCs in *C. brachyurus*, the only carcharhinid species in this dataset. Qualitatively, the macula neglecta of *C. brachyurus* was also the only where one patch of the macula was significantly larger than the other (see Fig. 5A), a feature previously reported in other carcharhinids (Tester et al. 1972; Corwin 1977). Importantly, this suggests that previous work published on carcharhinids might not be representative of the entire clade; rather, carcharhinid sharks may potentially possess a remarkably specialized macula neglecta in comparison to other species.

## Conclusions

Previous studies have demonstrated diversity in gross morphology of the inner ear of elasmobranchs, which may reflect the functional requirements of different species. Here, across nine shark species, we demonstrate interspecific variation in the inner ear HCs of sharks. Although results are based on limited data, shark species that feed in the water column tend to have greater HCD and HCT in vertically oriented maculae compared to benthic feeding species. Further, carcharhinids seemingly possess a remarkably large and specialized macula neglecta, which may not reflect the typical condition of this structure in elasmobranchs. Hair cell organization in sharks may vary with ecological characteristics, but a broader and more comprehensive examination of HC organization across a range of taxonomically and ecological diverse species is required to understand how variation in HC morphology relates to ecology in this clade.

## Supplementary Material

obad031_Supplemental_File

## Data Availability

The data that support the findings of this study will be made available from the corresponding author upon reasonable request.
