# Supplementary material for: Interspecific Variation in the Inner Ear Maculae of Sharks
Source: Integr Org Biol. 2023 Sep 4;5(1):obad031. doi: 10.1093/iob/obad031 (PMC10506894; doi:10.1093/iob/obad031)
Supplement: obad031_Supplemental_File [file obad031_supplemental_file.docx]

|  | **Macular area** | **HCD** | **HCT** |
| --- | --- | --- | --- |
| **Saccule** | 3.52 (0.10) | 0.86 (0.32) | 2.64 (0.14) |
| **Lagena** | 0.01 (0.93) | 0.15 (0.70) | 0.07 (0.78) |
| **Utricle** | 0.09 (0.77) | 2.30 (0.15) | 1.78 (0.18) |
| **Macula neglecta** | 0.01 (0.92) | 1.98 (0.16) | 0.95 (0.33) |

*Table S1*. F-statistics and p-values (in parentheses) from phylogenetic ANOVAs comparing mean HC organisation traits (pGLS residuals) between benthos and water column feeding species in the saccule, lagena, utricle, and macula neglecta.

*Table S2.* Raw data ranges for macular area, HCD, and HCT for each species examined in this study. Cells with a single value indicate parameters where only one observation was obtained.

|  |  | Macular area (mm^2^) | | |  |  |
| --- | --- | --- | --- | --- | --- | --- |
| **Species** | **n** | **Total length (cm)** | **Saccule** | **Lagena** | **Utricle** | **Macula Neglecta** |
| *A. vulpinus* | 2 | 148 - 152 | 6.5 - 7.0 | 7.7 - 10.0 | 8.2 - 9.9 | 0.7 |
| *C. brachyurus* | 2 | 73 - 78 | 4.3 | 2.6 - 3.0 | 7.9 | 13.1 - 14.0 |
| *G. galeus* | 3 | 48 - 62 | 2.3 - 2.8 | 0.9 - 1.3 | 3.2 - 4.0 | 0.5 - 0.6 |
| *I. oxyrinchus* | 2 | 100-121 | 2.4 - 2.6 | 1.8 - 1.8 | 2.0 - 2.0 | 0.4 - 0.6 |
| *S. zygaena* | 3 | 117 - 144 | 7.3 - 9.6 | 9.2 - 13.7 | 10.0 - 13.9 | 1.1 - 2.1 |
| *C. isabellum* | 3 | 49 - 62 | 0.9 - 1.3 | 0.6 - 0.9 | 0.8 - 1.0 | 0.3 - 0.4 |
| *M. lenticulatus* | 3 | 54 - 73 | 3.6 - 7.9 | 0.9 - 1.1 | 2.4 - 3.8 | 0.4 - 0.7 |
| *M. antarcticus* | 3 | 78 - 95 | 8.9 - 10.9 | 2.6 - 4.5 | 6.5 - 7.8 | 1.0 - 1.7 |
| *S. griffini* | 3 | 90 - 106 | 7.9 - 10.0 | 1.7 - 2.3 | 3.1 - 4.6 | 2.3 - 2.4 |
|  |  | HCD (#HCs/10,000 um^2^) | | |  |  |
| **Species** | **n** | **Total length (cm)** | **Saccule** | **Lagena** | **Utricle** | **Macula Neglecta** |
| *A. vulpinus* | 2 | 148 - 152 | 48.7 - 64.8 | 75.2 - 78.1 | 68.8 - 85.5 | 38.5 |
| *C. brachyurus* | 2 | 73 - 78 | 58.0 | 83.1 - 96.9 | 90.6 | 53.2 - 60.3 |
| *G. galeus* | 3 | 48 - 62 | 83.8 - 86.8 | 88.9 - 90.4 | 93.6 - 104.3 | 59.8 - 68.1 |
| *I. oxyrinchus* | 2 | 100-121 | 59.4 - 88.4 | 68.1 - 89.6 | 120.2 | 45.0 - 79.3 |
| *S. zygaena* | 3 | 117 - 144 | 24.8 - 36.6 | 63.7 - 83.0 | 70.1 - 100.3 | 16.2 - 23.4 |
| *C. isabellum* | 3 | 49 - 62 | 53.9 - 73.1 | 36.6 - 62.4 | 77.8 - 96.1 | 47.8 - 80.4 |
| *M. lenticulatus* | 3 | 54 - 73 | 40.3 - 80.8 | 69.8 - 84.1 | 85.0 - 130.4 | 19.7 - 63.3 |
| *M. antarcticus* | 3 | 78 - 95 |  |  |  |  |
| *S. griffini* | 3 | 90 - 106 | 31.2 - 45.8 | 14.9 - 24.0 | 70.6 - 93.3 | 25.4 - 26.5 |
|  |  |  | HCT |  |  |  |
| **Species** | **n** | **Total length (cm)** | **Saccule** | **Lagena** | **Utricle** | **Macula Neglecta** |
| *A. vulpinus* | 2 | 148 - 152 | 34,070 - 41,977 | 60,375 - 74,931 | 68,138 - 70,407 | 2,800 |
| *C. brachyurus* | 2 | 73 - 78 | 24693 | 24,583 - 24,948 - | 71,983 | 73,503 - 74,742 |
| *G. galeus* | 3 | 48 - 62 | 19,331 - 23,811 | 7,929 - 11,704 | 30,384 - 37,515 | 3,070 - 3,215 |
| *I. oxyrinchus* | 2 | 100-121 | 14,262 - 23,159 | 11,935 - 16,206 | 23,875 | 2,710 - 3,300 |
| *S. zygaena* | 3 | 117 - 144 | 9,757 - 34,934 | 63,269 - 113,878 | 81,919 - 115,556 | 2,495 - 3,689 |
| *C. isabellum* | 3 | 49 - 62 | 5,315 - 9,376 | 3,206 - 3,602 | 7,248 - 8,655 | 2,105 - 2,255 |
| *M. lenticulatus* | 3 | 54 - 73 | 19,143 - 40,112 | 7,649 - 8,334 | 23,738 - 31,245 | 807 - 2,870 |
| *M. antarcticus* | 3 | 78 - 95 |  |  |  |  |
| *S. griffini* | 3 | 90 - 106 | 25,094 - 45,569 | 2,994 - 5,544 | 21,762 - 42,577 | 6,094 - 6,172 |

**Supplementary data**

There were no significant differences between feeding zones in macular area (F_1,15_ = 0.44, p = 0.519), HCD (F_1,13_ = 0.08, p = 0.786), or HCT (F_1,13_ = 1.56, p = 0.234) residuals in the horizontally oriented macula (i.e., utricle). There were also no significant differences between feeding zones in macular area (F_1,15_ = 1.34, p = 0.265), HCD (F_1,13_ = 1.69, p = 0.217), or HCT residuals (F_1,13_ = 0.02, p = 0.888) in the ‘mixed’ oriented macula (i.e., saccule).
